# Remote bednet use monitoring to describe patterns of use and exposure to female *Anopheles* mosquitoes in an Ugandan cohort

**DOI:** 10.3389/fepid.2022.934557

**Published:** 2022-08-04

**Authors:** Paul J. Krezanoski, John Rek, Alex Musiime, Geoffrey Otto, Patrick Kyagamba, Jackson Asiimwe Rwatooro, Kelly Walters, Alina Romanel, Emmanuel Arinaitwe, Joaniter I. Nankabirwa, Chris J. Drakeley, Moses Kamya, Grant Dorsey

**Affiliations:** ^1^University of California, San Francisco, San Francisco, CA, United States; ^2^Opportunity Solutions International, San Francisco, CA, United States; ^3^Infectious Diseases Research Collaboration, Kampala, Uganda; ^4^US President's Malaria Initiative, Kampala, Uganda; ^5^Makerere University College of Health Sciences, Kampala, Uganda; ^6^London School of Hygiene and Tropical Medicine, London, United Kingdom

**Keywords:** malaria control, insecticide-treated bednets, electronic bednet use monitoring, *Anopheles* mosquito, human behavior

## Abstract

**Background:**

Long lasting insecticide-treated bednets (LLINs) are the most widely used tool for preventing malaria. There has been a plateau in progress in the highest burden African countries since 2015, leading to questions about the effectiveness of LLINs. In this study, remote LLIN use monitors were deployed in a cohort in Eastern Uganda to explore how LLIN use interacts with mosquito exposure.

**Methods:**

The SmartNet study included 20 households from May to October 2019. SmartNet devices recorded, every 15 min, whether an LLIN was unfurled or folded up. Unannounced visits were used to assess SmartNet accuracy. Risk factors associated with poor LLIN use were assessed using generalized linear equations. Female *Anopheles* exposure was estimated by combining hourly probabilities of exposure from human landing catches and measures of density from biweekly CDC light traps in participants rooms. Mosquito exposure averted by LLINs was quantified using SmartNet measurements and age-related differences were estimated using generalized linear equations, adjusting for relevant covariates and household clustering.

**Results:**

96 individuals contributed 5,640 SmartNet observation nights. In 126 unannounced visits, SmartNet had an area under the curve of 0.869 in classifying whether the LLIN was up or down. The rate of non-use was 13.5% of nights (95% CI: 12.6–14.3%). Compared to children under 5, non-use was 1.8 times higher (95% CI: 1.6–2.1; *p* < 0.001) in children 5–15 years and 2.6 times higher (95% CI: 2.2–3.1; *p* < 0.001) in participants aged 15– <30years. There was no difference between children under 5 years and adults > 30 years. LLIN use averted 50.3% of female *Anopheles* mosquito exposure (95% CI: 40.0–60.0%), with decreasing point estimates of efficacy across age groups: from 61.7% (95% CI: 42.6–80.7%) in children under 5 years to 48.0% (95% CI: 29.1–66.8%) in adults over 30.

**Conclusions:**

Objective monitors are accurate and can feasibly be deployed to obtain data about LLIN use. LLINs provided protection from only 50% of female *Anopheles* mosquito exposure in this cohort and protection was dependent upon age. In assessing the role of LLINs in malaria prevention it is crucial to consider the dynamics between mosquito exposure and LLIN use behaviors.

## Introduction

Insecticide-treated bednets (ITNs) and more recently long-lasting insecticidal-treated bednets (LLINs) are the most widely used tool for preventing malaria and make up a significant share of funding for malaria prevention in sub-Saharan Africa (1). Randomized controlled trials from the 1990s demonstrated that ITNs were highly effective (2) and it has been estimated that between 2000 and 2015 the incidence of malaria decreased by 40% in sub-Saharan Africa, with ITNs responsible for 68% of cases averted (3). Since 2015, however, progress has stalled and even reversed course in some of the highest burden countries in Africa (1). There is concern that increasing vector resistance to pyrethroid insecticides used in LLINs is contributing to this trend (4, 5), but there is limited evidence that insecticide resistance is compromising the effectiveness of LLINs (6). As a result, other factors threatening the effectiveness of LLINs should be considered, including recent evidence of changes in mosquito biting behavior and how people use their LLINs (7–9). To better understand these, there is an increasing need for tools that facilitate studies of the dynamic interaction between mosquito exposure and human behaviors, including LLIN use, as they relate to malaria risk.

LLIN use is most commonly measured through surveys that ask individuals whether or not they slept under an LLIN the prior night. This subjective, summary, question is easy to administer and useful for assessing trends in LLIN use. However, there is evidence that reported LLIN use over-estimates actual use (10). In addition, assessing LLIN use as a simple binary measure provides only limited insight into the essential interaction at the core of an LLINs' main malaria prevention function: alignment between the timing of protection and the timing of exposure to mosquitoes that transmit malaria.

Compared to self-reporting methods, new tools for more reliably measuring LLIN use at higher resolution have been developed in recent years (11, 12). These tools have been found in small studies to be acceptable to local populations in Uganda (13, 14) and feasible to deploy (15), yet there remain unanswered questions about their accuracy in real-life settings and how their use might alter typical LLIN use behaviors. Furthermore, very few studies exist that objectively examine how LLINs are actually used throughout the night (16, 17), and no study has yet explored risk factors associated with LLIN use measured by objective monitors, nor quantified how objectively measured LLIN use overlaps with exposure to female *Anopheles* mosquitoes.

In this study, objective LLIN use monitors were deployed in a cohort of individuals of all ages undergoing surveillance of reported LLIN use and mosquito exposure in Eastern Uganda. LLIN use was quantified, and risk factors associated with poor LLIN use were assessed. Unannounced spot checks were performed to assess the accuracy of the objective monitoring device. Hourly female *Anopheles* exposure was estimated, and the share of mosquito exposure averted by LLINs quantified after accounting for objectively and precisely measured LLIN use. The goals of this approach were to uncover new insights into how LLINs are used in practice and advance knowledge of how use of LLINs interacts with mosquito exposure to prevent malaria in endemic settings.

## Methods

### Study setting and population level malaria control interventions

This sub-study (termed “SmartNet”) was nested within a larger cohort and entomological surveillance study conducted in Nagongera sub-county, Tororo District, Uganda from October 2017 to October 2019. Before 2013, malaria control in Tororo was limited to the distribution of LLINs through antenatal care services, promotion of intermittent preventive treatment during pregnancy, and malaria case management with artemisinin-based combination therapy. In November 2013, universal distribution of free LLINs was conducted as part of a national campaign, and a similar campaign was repeated in May 2017. Indoor residual spraying (IRS) with the carbamate bendiocarb was first initiated in December 2014–January 2015, with additional rounds administered in June–July 2015 and November–December 2015. In June–July 2016, IRS was administered with the organophosphate pirimiphos-methyl (Actellic), with repeated rounds in June–July 2017, June–July 2018, and March–April 2019. Implementation of these vector control interventions was associated with a marked decline in transmission intensity with the annual entomological inoculation rate declining from 238 infective bites per person per year pre-IRS to 0.43 after 4–5 years of IRS (18).

### Parent cohort study and entomological surveillance

Details of the parent cohort study and entomological surveillance have been published previously (18, 19). Briefly, in October 2017 all permanent residents of 80 randomly selected households within Nagongera subcounty were enrolled. The cohort was dynamic such that over the course of the study, any permanent residents who joined the household were enrolled and individuals no longer residing in the household were withdrawn. All household participants were given access to an LLIN at the time of enrollment. Participants were followed through October 2019.

Mosquito collections were conducted every 2 weeks in all households. In each room where study participants slept, a miniature CDC light trap (Model 512; John W. Hock Company, Gainesville, FL) was positioned 1 m above the floor at 7 p.m. and collected 7 a.m. the following morning to quantify the number of female *Anopheles* captured per room per night. On the morning of the biweekly CDC light trap collections, the following data were also collected on all household members who slept in the house the prior night: (1) whether or not they slept under an LLIN (yes or no), (2) time getting into bed, (3) time getting out of bed, and (4) the room and sleeping area where they slept.

Human landing catches (HLC) were performed every 4 weeks from November 2017 to October 2018 in 8 non-cohort households randomly selected from the same study area (19). In brief, two field workers were stationed indoors with exposed legs and they collected mosquitoes using aspirators and flashlights from 6 p.m. until 6 a.m. the following morning. Mosquitoes were labeled with the hour of capture, and females of the *Anopheles* species were identified and stored for future analysis.

### SmartNet study participant selection and follow-up procedures

**Figure 1:**
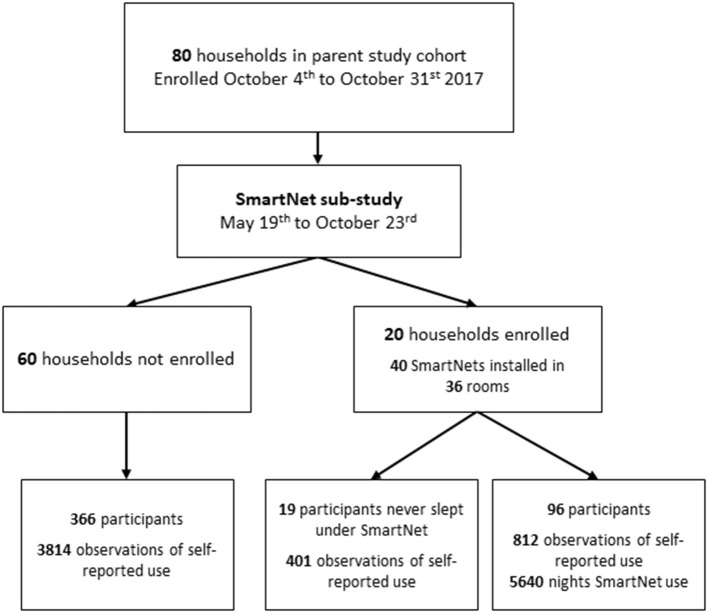
Flow diagram of households and participants.

The SmartNet study began enrollment in May 2019 and continued follow up until the end of the parent cohort study in October 2019. Figure 1 summarizes the participant flow from the parent study to the SmartNet sub-study. Given limitations on the number of monitoring devices available, a sub-sample of 20 households from the parent study were chosen to participate. Households were purposefully chosen that were reported by the field team to have LLINs hanging above most sleeping areas in the household and were reporting regular LLIN use in the biweekly surveys. After providing informed consent, each regularly used sleeping area with a hanging bednet was replaced with an objective monitoring SmartNet in participating households. Sleeping areas that were infrequently used or did not have a bednet hanging above them, and individuals using those sleeping areas, were not subject to SmartNet monitoring.

SmartNets have been described in detail elsewhere (11, 15), but, in brief, they are World Health Organization-approved rectangular LLINs that use conductive fabric interwoven into the sides and top of the net to determine whether the bednet is unfurled or folded up for storage. Every 15 min the SmartNet records the state of the net (up or down) with a timestamp on a removable SD memory card. At the already occurring biweekly study visits, the SD card containing the SmartNet data was retrieved and identified with the household identification number and the room number/sleeping area over which it was hanging. Using the reported room number/sleeping area for each individual, the SmartNet data from the two previous weeks was then be matched to each individual who slept under a monitored sleeping area.

### Variable definitions and procedures

SmartNet accuracy was assessed using unannounced visits to households during which researchers observed and recorded whether each SmartNet in the household was folded up or unfurled. The researchers planned to make four unannounced visits to each household, two between 8 p.m. and 9 p.m. and two between 6 a.m. and 7 a.m. A total of 160 observations were planned (4 each for 40 SmartNets), but occasionally these visits were unsuccessful due to participants not being home. Overall, a total of 126 unannounced observations were completed, with corresponding SmartNet measures successfully visualized: 65 at night and 61 in the morning. In addition, there were four occasions where the SmartNet device detected a change (from up to down, for example) at the same time that the researchers approached the house. In these cases where there was a discrepancy between the observed state of the net and the SmartNet record, the record was adjusted to match the state of the SmartNet before the switch was made. SmartNet accuracy was determined by using the observed state of the SmartNet as the reference against which to compare the SmartNet measurement of whether the bednet was up or down. An additional analysis was performed that instead dropped the observations with the discrepancies and, finding no significant change in the overall accuracy, the main method was retained.

To assess whether objective bednet monitoring itself may have had an impact on reported LLIN use, individual reported LLIN use after the start of SmartNet deployments was compared in three different groups: (1) individuals in 60 households not enrolled in the SmartNet sub-study, (2) individuals in the 20 SmartNet households who slept in areas not covered by a SmartNet and (3) individuals who slept under SmartNets.

To overlap with the timing of HLCs, the observation period for SmartNet-measured LLIN use was from 6 p.m. until 6 a.m. A missed night of use was defined as no SmartNet-measured use during this observation period. The rate of nights without use for each individual was defined as the number nights with no use divided by the nights of observation.

The number of hours of use per night was compared using histograms across four different methods of assessing LLIN use. This comparison was restricted to nights where there was a reported measure of individual LLIN use and bedtimes from the biweekly surveys. Since no one in the cohort reported waking up before 6 a.m., the analysis below uses only reported bedtimes and not waking times. The first method for calculating duration of LLIN use utilized reported use the prior night alone, attributing 12 h of LLIN use if the individual reported using the bednet and 0 h if the individual reported not using the bednet. The second method counted hours of use by using reported use plus incorporating reported bedtimes from the most recent biweekly survey. The third method used only the SmartNet record for the night summarized at hourly resolution. The fourth method used both the SmartNet record and reported bedtimes summarized at hourly resolution. In addition, the estimated proportion of LLINs in use per hour was calculated using each of the methods described above that provided data on hourly use (second through fourth methods).

**Figure 2:**
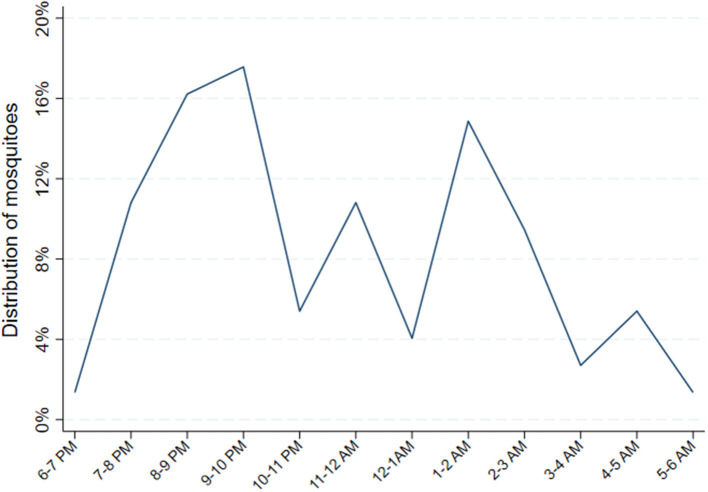
Distribution of female *Anopheles* mosquitoes from indoor human landing catches. Probability distribution of *Anopheles* exposure calculated by pooling the total number of female *Anopheles* captured from 48 catches performed indoors from 6 p.m. to 6 a.m. in 8 households, geographically proximate to the main cohort households, where HLCs were conducted from May through October 2018. Then, for each hour, the number of female *Anopheles* captured that hour was divided by the total number of female *Anopheles* captured throughout the entire night. This resulted in an hourly probability distribution of indoor biting female *Anopheles*.

Relative hourly exposure to female *Anopheles* mosquitoes was estimated for each individual for each night between 6 p.m. and 6 a.m. according to the following procedure. First, total nightly mosquito exposure was estimated from the biweekly CDC LT data. For the nights when CDC light traps were performed, there were direct measures of the number of female *Anopheles* captured in the room where each individual slept. For nights when there was no CDC light trap performed, exposure was estimated by applying the most recent CDC light trap yield. Next, the HLC data during the same calendar months from the year prior (May to October 2018) was used to obtain a summary estimated distribution of indoor biting female *Anopheles* by hour. This was achieved by pooling the total number of female *Anopheles* captured indoors from 6 p.m. to 6 a.m. in the 8 households where HLCs were conducted across the 4 months. Then, for each hour, the number of female *Anopheles* captured that hour was divided by the total number of female *Anopheles* captured throughout the entire night. This resulted in an hourly probability distribution of indoor biting female *Anopheles* (Figure 2). Finally, hourly exposure was estimated for each individual for each night by applying the probabilities of exposure by hour from the HLC data to the total number of estimated female *Anopheles* mosquito exposure for the night from the CDC LT data. The estimated nightly quantity of female *Anopheles* exposure from the CDC light traps, therefore, was distributed throughout the night hours according to the hourly probabilities of exposure estimated from the HLCs.

The method above utilizes only indoor biting female *Anopheles* from the HLCs and assumes, conservatively, that individuals are indoors beginning at 6 p.m. Outdoor HLCs were performed on the same nights around the same households as the indoor catches except that outdoor collections were limited to 6 p.m. until 12 a.m. In a separate sensitivity analysis, we also incorporated outdoor biting by assuming, on the other extreme, that individuals were outdoors up until the moment they reported going to bed. This method resulted in even more pronounced peaks in the probability distribution of *Anopheles* exposure earlier in the night (Supplementary Figures S1–S3). To achieve an estimate of hourly *Anopheles* exposure, the probability of exposure per hour was utilized as above. Additionally, since outdoor density was consistently higher than indoor in the HLCs, the total number of *Anopheles* caught per hour as estimated by the CDC LTs was upweighted by the average factor that the outdoor HLCs were greater than indoor in that hour. For example, outdoor caught *Anopheles* were 3.75 × greater in number than the indoor HLCs from 7 to 8 p.m. over the 48 nights, so the estimated quantity of *Anopheles* from the CDC LT data for 7–8 p.m. was augmented by a factor of 3.75. This method led to a much lower estimate of the protection afforded by LLIN use in the methods that incorporated reported bedtimes (Supplementary Figure S4). Since data on the timing of when participants were indoors vs. outdoors prior to going to bed was unavailable, the previous, clearly conservative, estimate that all individuals were indoors from 6 p.m. until 6 a.m. was adopted for the main analysis.

Estimates for the protection afforded by LLIN use was assessed by summing the relative number of female *Anopheles* each individual could be exposed to indoors each night and, assuming 100% protection when sleeping under an LLIN, subtracting the mosquito exposure during the hours with measured LLIN use according to the four methods above. The relative proportion of female *Anopheles* exposure averted due to LLIN use per night was calculated by dividing the estimated number of mosquitoes to which an individual would be exposed accounting for LLIN use by the estimated mosquito exposure assuming no LLIN use.

### Statistical analysis

For summary statistics, means and standard deviations were reported for normally distributed continuous variables such as age. Medians and interquartile ranges were reported for non-normally distributed variables such as the number of residents in the household. Receiver operating characteristics, a 2 × 2 table and the area under the curve (AUC) was calculated for the comparison of SmartNet-measured state of the LLIN to the observed LLIN state as the reference. The total number of nights with no SmartNet-measured LLIN use was calculated for each individual. Risk factors associated with non-use were assessed using bivariate and multivariate generalized estimating equations assuming a Poisson distribution with the count of nights without use as the outcome and the number of nights of observation as the exposure. Covariates included age, gender, mosquito exposure. Following trends in the data and to aid in interpretation, covariates were separated into categories. Age was separated into four categories: under 5 years, five to under 15 years, 15 to under 30 and over 30 years of age. Mosquito exposure based on the mean number of female *Anopheles* mosquitoes captured over the study period from biweekly CDC light trap collections in each participant room was stratified into three categories: <2 mosquitoes on average, 2 to <6 and >6 mosquitoes. Analyses accounted for clustering of individuals within the same household, assumed an exchangeable covariance structure and are reported as rate ratios (RR) with 95% confidence intervals (CIs). To compare the four different methods of assessing LLIN use the sample was restricted to the 392 nights among 95 participants when reported LLIN use was available. The proportion of female *Anopheles* mosquito exposure averted was calculated by dividing the sum of estimated mosquito exposures according to the four methods of assessing LLIN use above by the estimated number of mosquito exposures without LLIN use and 95% CIs were calculated. In separate analyses, using the full sample, generalized estimating equations assuming a Poisson distribution with individual counts of *Anopheles* exposures across the study as the outcome were used to obtain marginal estimates by age category for mosquito exposure with and without LLIN use, again using the number of nights of observation as the exposure and accounting for clustering at the household level. These analyses also were adjusted for gender and the number of people sleeping in the room. The proportion of *Anopheles* exposures averted, with 95% CIs, was calculated for each age group by dividing the marginal estimated count of mosquito exposures with LLIN use by the estimated exposure without LLIN use.

## Results

### Cohort demographic characteristics

Twenty households were enrolled in the SmartNet sub-study and their characteristics were generally comparable to the other 60 households in the cohort according to the number of residents, sleeping rooms and sleeping areas (Table 1). A higher proportion of SmartNet households tended to be from the highest wealth tertile compared to the non-SmartNet households (45 vs. 28%). Of the 115 participants in SmartNet households, 96 participants spent at least one night under a SmartNet. Age and gender characteristics were also generally comparable between participants monitored by SmartNet and the 385 individuals not monitored by SmartNet (19 from SmartNet households and 366 from other households).

**Table 1:** Baseline demographic characteristics at SmartNet enrolment.

**Household characteristics**	**Enrolled in** **SmartNet**	**Not enrolled**
	***N*** **=** **20**	***N*** **=** **60**
Residents, median (IQR)	6 (2)	6 (2)
**Wealth tertile**, ***n*** **(%)**		
Lowest	4 (20.0%)	25 (41.7%)
Middle	7 (35.0%)	18 (30.0%)
Highest	9 (45.0%)	17 (28.3%)
Rooms for sleeping, median (IQR)	2 (1)	2 (1)
Sleeping areas, median (IQR)	3 (1)	3 (2)
LLIN ownership, *n* (%)	20 (100%)	60 (100%)
LLINs per sleeping area, mean (SD)	0.5 (0.2)	0.4 (0.1)
**Individual characteristics**	**Monitored by SmartNet**	**Not monitored**
	***N*** **=** **96**	***N*** **=** **385**
Female, *n* (%)	52 (54.2%)	201 (52.2%)
Age in years, mean (SD)	18.0 (16.1)	17.1 (16.3)
**Age categories**, ***n*** **(%)**		
<5 years	25 (26.0%)	85 (22.1%)
5 to <15 years	34 (35.4%)	172 (44.7%)
15 to <30 years	10 (10.4%)	42 (10.9%)
Over 30 years	27 (28.1%)	86 (22.3%)

IQR, interquartile range.

### Field assessment of SmartNet accuracy based on visual observations

**Figure 3:**
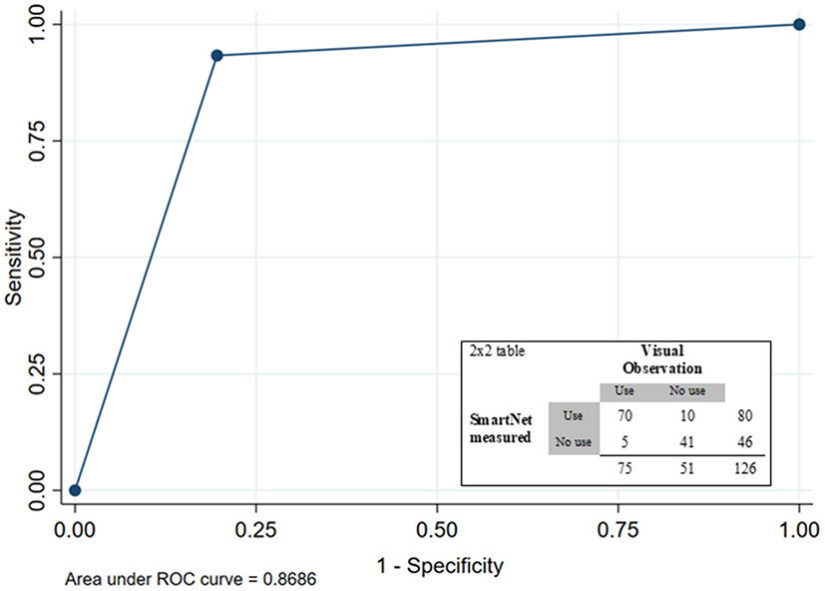
Receiver-operating curve (ROC) and 2 × 2 table for SmartNet-measured LLIN state based on visual observation as reference.

Based on the unannounced visits, yielding 126 visual assessments of the state of the LLIN as the reference and SmartNet measurements as the comparison, the area under the curve (AUC) was 0.869 (Figure 3). SmartNet tended to be more accurate in detecting LLINs that were unfurled for use 93.3% (70/75) than LLINs that were folded up 80.4% (41/51). Overall SmartNet accuracy was 88.1% for correctly classifying the state of the LLIN compared to visual assessments.

### Effect of bednet monitoring on LLIN use behaviors

**Figure 4:**
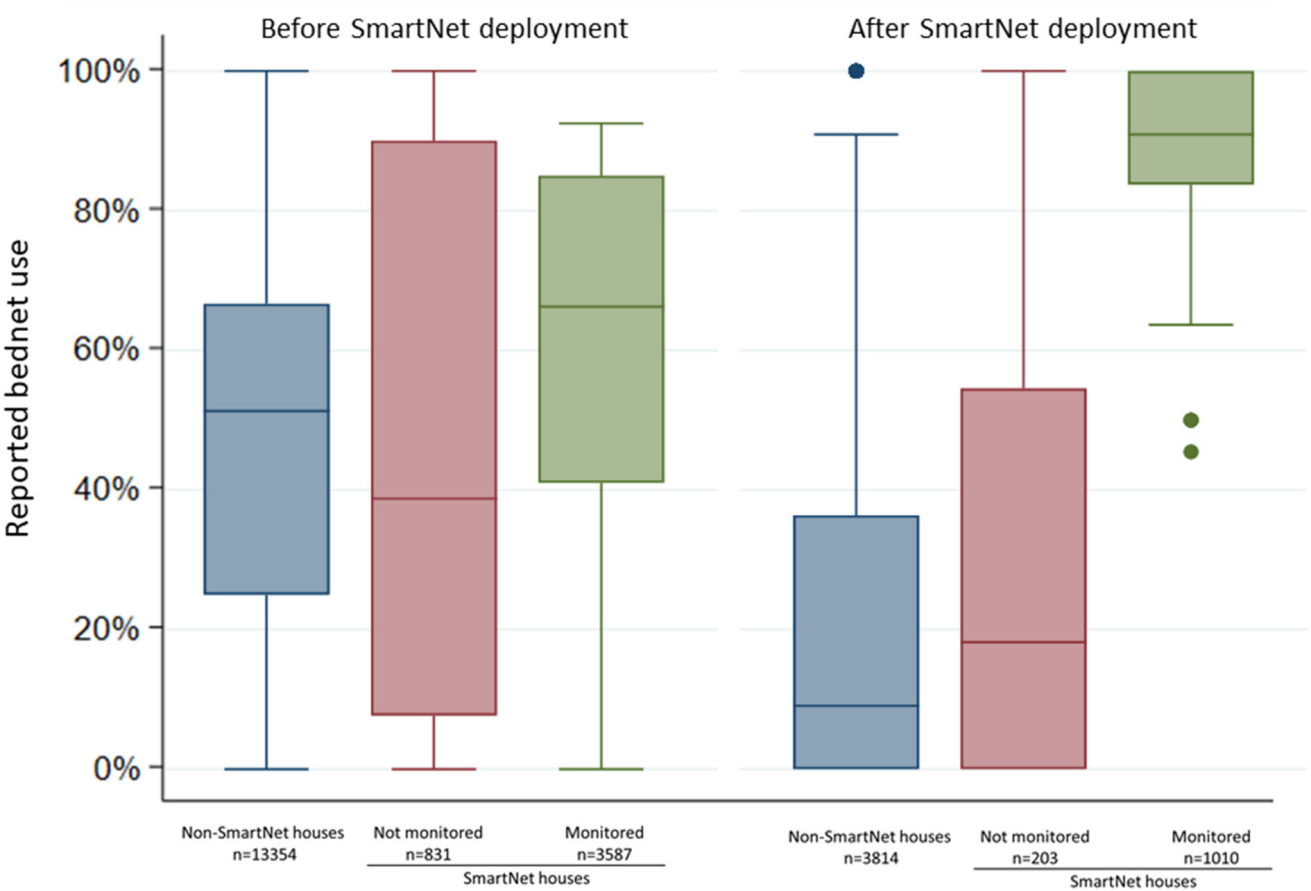
Comparison of individual reported bednet use at biweekly surveys before and after SmartNet deployment stratified by SmartNet monitoring status. Box plot where lines represent the median, boxed areas represent the interquartile range (IQR), whiskers represent the “minimum” and “maximum” defined as ± 1.5 * IQR and points represent outliers beyond the minimum or maximum. *N* values represent the number of measures of reported LLIN use per group.

Comparing reported individual LLIN use at the biweekly surveys, individuals who were monitored by SmartNet had markedly higher reported LLIN use compared to the other groups during the period of SmartNet deployment from May to October 2019 (Figure 4). Mean reported LLIN use for 96 monitored individuals across 1,010 observations was 85.5% (95% CI: 83.5.0–87.6%) compared to 20.9% (95% CI: 19.7–22.1%) from 203 observation for 19 individuals in the same households who were not monitored and 14.5% (95% CI: 9.2–19.7%) from 3,814 observations for 366 individuals who were not in SmartNet households.

### Factors associated with not using LLINs

Using SmartNet measurements over 5,640 observation nights, the overall rate of non-use was 13.5% (95% CI: 12.6–14.3%). The rate of non-use increased with increasing time since enrollment, from 3.3% (2.0–4.7%) in the first month, 8.8% (7.6–10.0%) in months 2–3 and 19.3% (17.9–20.8%) in months 4–5. Significant associations were found between a variety of covariates and the rate of non-use in the multivariate model that accounted for clustering at the household level (Table 2). Compared to children under 5 years of age, the non-use rate was 1.8 times higher (95% CI: 1.6–2.1; *p* < 0.001) in children five to under 15 years and 2.6 times higher (95% CI: 2.2–3.1; *p* < 0.001) in participants aged 15 to under 30 years. There was no statistically significant difference between the non-use rate in children under five and adults 30 years and older (*p* = 0.351). The rate of non-use was 1.2 times higher in males compared to females (95% CI: 10.8–33.6%; *p* < 0.001). Individuals experiencing lower levels of mean nightly female *Anopheles* mosquito exposure over the study period had higher non-use rates. For example, compared to individuals with a mean nightly mosquito exposure of 6 or more mosquitoes, individuals that had <2 mosquito exposures per night on average had 2.4 times the rate of non-use (95% CI: 1.8–3.1; *p* < 0.001).

**Table 2:** Risk factors associated with not using a bednet as measured by SmartNet.

					Bivariate*		Multivariate*	
**Risk factors**	**Number of participants**	**Nights of observation**	**Nights without use**	**Crude rate of non-use**	**Adjusted RR (95% CI)**	***p*-value**	**Adjusted RR (95% CI)**	***p*-value**
**Age category**								
Under five	25	1,363	142	10.5%	Reference		Reference	
5 to <15	34	2,144	348	16.2%	1.9 (1.7–2.3)	<0.001	1.8 (1.6–2.1)	<0.001
15 to <30	10	560	159	28.4%	2.5 (2.1–3.0)	<0.001	2.6 (2.2–3.1)	<0.001
30–57	27	1,573	110	7.0%	0.9 (0.8–1.1)	0.351	1.0 (0.9–1.1)	0.739
**Gender**								
Female	52	2,992	374	12.5%	Reference		Reference	
Male	44	2,648	385	14.5%	1.3 (1.2–1.4)	<0.001	1.2 (1.1–1.3)	<0.001
**Mosquito exposure** ^†^								
6 and greater	19	784	70	8.2 %	Reference		Reference	
2 to <6	33	1,699	285	14.4%	1.4 (1.1–1.9)	0.008	1.3 (1.0–1.7)	0.024
<2	44	2,398	404	14.4%	2.5 (1.9–3.4)	<0.001	2.4 (1.8–3.1)	<0.001

CI, Confidence Interval; RR, rate ratio.

^*^Adjusted rate ratios estimated with generalized estimating equations using Poisson regression and accounting for clustering at the household level assuming an exchangeable within-group correlation structure.

^†^Mean number of anopheles mosquitoes captured from participant sleeping room every 2 weeks using overnight CDC light traps during study period.

### Comparison of four methods of quantifying hours of LLIN use

**Figure 5:**
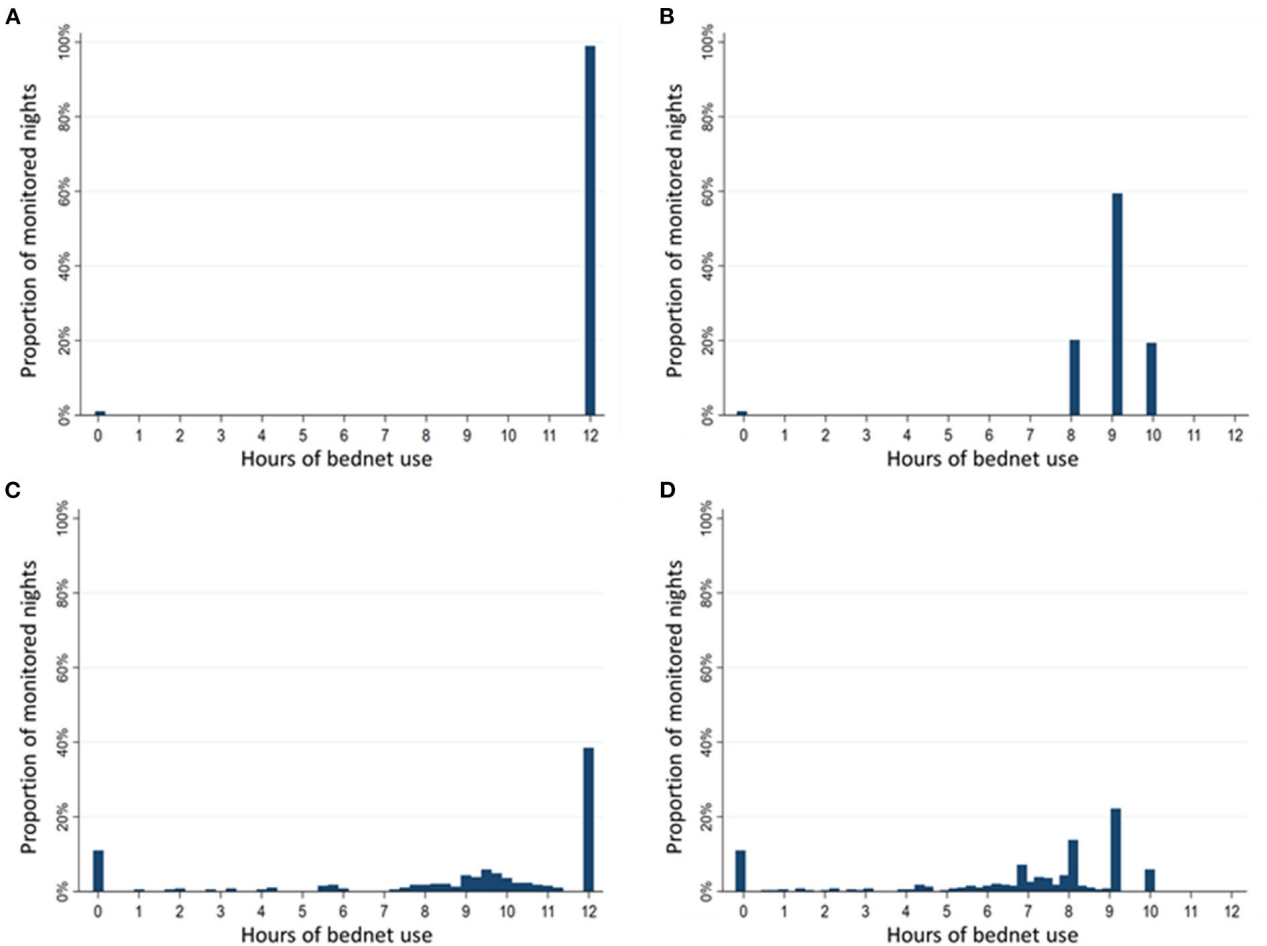
Comparison of duration of bednet use per night by measurement method. Sample restricted to 392 nights with reported use and assessed over 95 participants with reported use data. **(A)** Histogram of hours of use based on reported bednet use alone. **(B)** Histogram of hours of use based on reported bednet use plus reported bedtimes. **(C)** Histogram of hours of use based on SmartNet-measured bednet use alone. **(D)** Histogram of hours of use based on SmartNet-measured bednet use plus reported bedtimes.

Estimated duration of LLIN use per night differed substantially depending on the method used to assess the duration of use. The distribution of hours of LLIN use were compared using histograms of use among 95 participants (one participant was excluded due to incomplete data) over 392 nights of observation when there were direct measures of reported LLIN use, reported bedtimes and SmartNet measurements (Figure 5). Using only reported measures of LLIN use and bedtimes, there is a clustering of estimated hours of use reflecting no use at all (0 h) or the reported bedtime (8 p.m. until 6 a.m., for example, equals 10 h) (Figure 5B). Using SmartNet data alone provides an estimated rate of non-use of 13% (Figure 5C), but this likely overestimates the duration of use because it assumes 12 h of use if the LLIN was measured as unfurled continuously from 6 p.m. to 6 a.m. Combining SmartNet data with reported bedtimes provides the most plausible and reticulated estimates of hourly use (Figure 5D). According to these four methods, the estimated mean duration of LLIN use in the restricted sample with direct measures of reported use were: 11.9 h (95% CI: 11.8–12.0) using reported LLIN use alone, 8.9 h (95% CI: 8.8–9.0) using reported LLIN use and bedtimes times, 8.9 h (95% CI: 8.5 to 9.3) using SmartNet data alone and 6.7 h (95% CI: 6.4–7.0) using SmartNet data plus reported bedtimes times.

**Figure 6:**
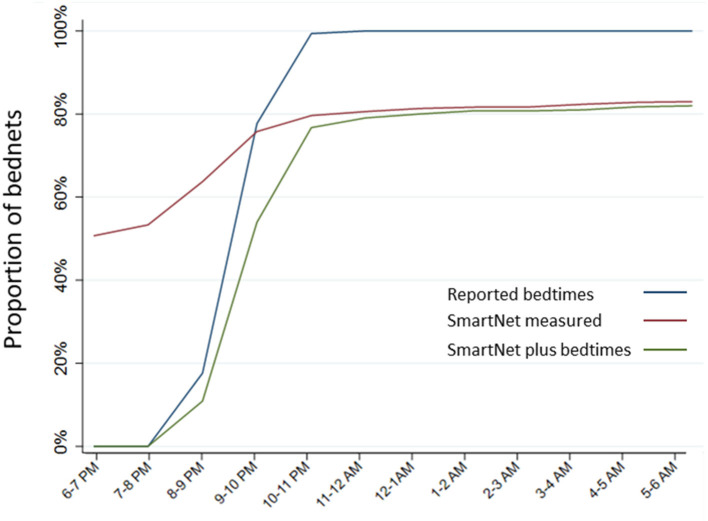
Estimated proportion of LLINs in use per hour by measurement method. Estimates of hourly LLIN use made for each of the three measurement methods that provide data on hourly use: reported use plus bedtimes, SmartNet-measured bednet use alone and SmartNet-measured bednet use plus reported bedtimes.

The estimated proportion of bednets in use per hour was compared across the three methods above that provide estimates of hourly use: reported use plus bedtimes, SmartNet alone and SmartNet combined with bedtimes. Estimating the timing of LLIN use with reported bedtimes only there is a tendency to over-estimate use later in the evening. Using SmartNet data alone, on the other hand, tends to over-estimate use earlier in the night (e.g., before 9 p.m.) when participants are not yet sleeping under an unfurled LLIN. Combining reported bedtimes and SmartNet data leads to the most precise estimates of hourly LLIN protection (Figure 6).

### Comparison of methods for quantifying female *Anopheles* mosquito exposure averted by LLIN use

**Figure 7:**
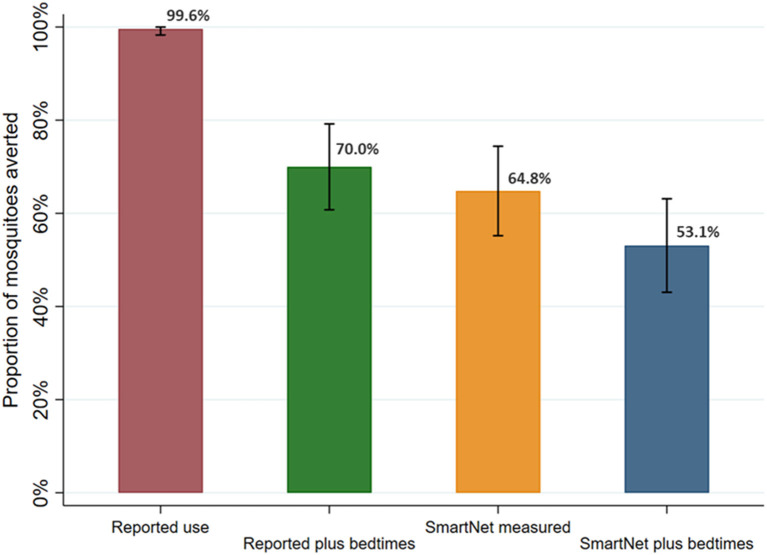
Estimated proportion of female *Anopheles* mosquito exposure averted from bednet use by measurement method. Sample restricted to 392 nights with reported use and assessed over 95 participants with reported use data. Bars represent 95% confidence intervals around labeled means.

Continuing with the sample restricted to 392 nights where there were direct measures of reported LLIN use and bedtimes times, the estimated proportion of female *Anopheles* exposures from 6 p.m. to 6 a.m. averted by LLIN use was calculated and compared (Figure 7). These 392 nights also had direct measures, *via* CDC light traps, of female *Anopheles* mosquito density the prior night. Given the high rate of reported use, using reported LLIN use alone led to an estimated 99.6% (95% CI: 98.3–100%) of mosquito exposures averted. Using reported LLIN use and bedtimes, an estimated 70.0% (95% CI: 60.8–79.2%) of mosquitoes were averted. Using SmartNet data alone led to an estimate of 64.8% (95% CI: 55.2–74.4%) of mosquitoes averted. Finally, using SmartNet data and reported bedtimes, an estimated 53.1% (95% CI: 43.0–63.1%) of female *Anopheles* mosquito exposures were averted due to LLIN use in this restricted sample.

Of note, in the admittedly extreme sensitivity analysis adding outdoor biting data from the HLCs described above, the proportion of female *Anopheles* averted due to bednet use declined substantially using the methods that allowed for estimates of outdoor exposure (Supplementary Figure S4). For example, incorporating estimates of outdoor exposure and using reported bedtimes and SmartNet data resulted in an estimated 17.0% (95% CI: 9.5–24.6%) of female *Anopheles* exposure averted with bednet use.

### Female *Anopheles* exposure averted due to LLIN use in full sample and age-related differences

**Figure 8:**
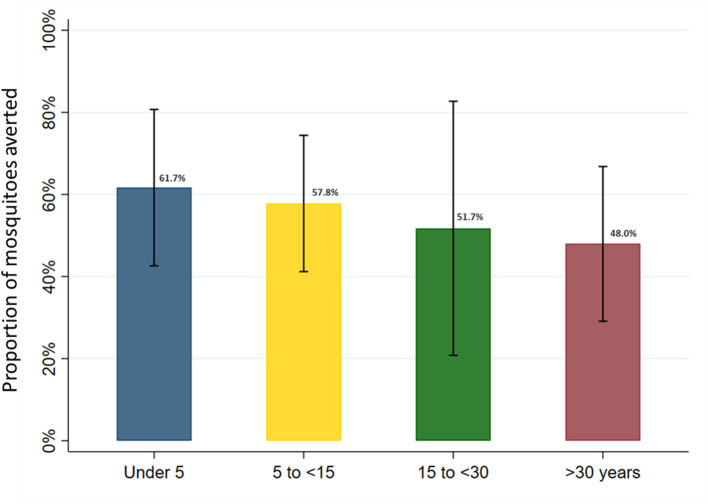
Estimated proportion of female *Anopheles* mosquito exposure averted from bednet use by age category in full study sample. Marginal estimates calculated from generalized estimating equations using Poisson regression and adjusted for gender and the number of people sleeping in the room. Models account for clustering at the household level and assume an exchangeable within-group correlation structure. Bars represent 95% confidence intervals around labeled means.

In the full sample of 5,640 nights of observation, the human biting rate was 4.1 mosquitoes per night (95% CI: 2.0–8.1). Overall, mean nightly female *Anopheles* mosquito exposure adjusted for LLIN use, according to the SmartNet plus the most recent bedtimes method, was 2.0 per night (95% CI: 0.7–3.4). LLIN use across all age groups in this cohort, therefore, averted an estimated 50.3% of female *Anopheles* mosquito exposure (95% CI: 40.0–60.0%). Given age-specific differences in baseline mosquito exposure and LLIN use patterns, heterogeneity was present between age groups in the point estimates of the protective efficacy of LLINs (Figure 8). After adjusting for gender, the number of people sleeping in the room and household clustering, LLIN use averted 61.7% (95% CI: 42.6–80.7%) of female *Anopheles* in under 5 year olds, 57.8% (95% CI: 41.2–74.4%) in 5 to under 15 year olds, 51.7% (95% CI: 20.8–82.7%) in 15 to under 30 year olds and 48.0% (95% CI: 29.1–66.8%) in adults over 30 years of age. While the trend in the point estimates suggest a difference in protective efficacy, the overlap in the 95% confidence intervals indicate a lack of power to conclude a statistically significant difference between the age groups.

## Discussion

In this cohort from Eastern Uganda, LLIN use measured with an objective LLIN use monitor and accounting for reported bedtimes was estimated to provide protection against only 50% of female *Anopheles* mosquito exposure. This limited protection was achieved despite very high reported LLIN use in this cohort (99.6%), and similarly high LLIN use objectively confirmed by the electronic monitor (86.5%). Perhaps unsurprisingly, due to underlying behavior differences, point estimates of the effective protection of LLINs varied by age group, decreasing from an estimated 62% in children under 5 years of age to 48% in adults over 30 years.

Multiple studies have estimated the protective efficacy of bednets using measures of hourly mosquito density and applying reported measures of bednet use, but this study is the first to use objective monitoring of hourly bednet use. The estimates of LLIN protection from this study are lower than those from recent studies in Benin (80–87%) (20) and Burkina Faso (80–85%) (21), but are generally in line with those from Tanzania (38–70%) (22, 23) and Kenya (51%) (24). Differences may be attributed to variations in local LLIN use behaviors, local variations in the timing of mosquito biting or differences in methods. Without a direct measure of when individuals were indoors vs. outdoors in this study, the conservative estimate that all individuals were indoors beginning at 6 p.m. was utilized. As demonstrated in a sensitivity analysis, incorporating outdoor biting would further decrease the apparent efficacy of LLIN use in this cohort (Supplementary Figure S4). Although it is important to point out that this finding is driven by significantly higher outdoor biting rates compared to indoor in this study, and this might not be the case in other settings. More precise measures of female *Anopheles* exposure could be obtained by using objective monitors of LLIN use as in this study and adding measures of indoor/outdoor movements before bedtimes, either reported or objectively monitored, as has been done in other studies (22). These studies of the protection afforded by LLINs provide crucial evidence that the alignment between the timing of changes in mosquito exposure and individual behaviors is an important determinant of malaria risk. This interplay between human and vector behaviors may well be more important in terms of LLIN effectiveness than the focus on insecticide resistance that has driven much of the efforts to improve LLIN effectiveness in recent years (7).

The rate of objectively measured non-use of LLINs in this study was higher among school age children (1.8 ×) and young adults (2.6) compared to children under 5 years and adults over 30. In addition, rates of non-use tracked with overall female *Anopheles* mosquito exposure, with individuals exposed to fewer mosquitoes more likely to miss a night of LLIN use. These findings are generally in line with findings from reported LLIN use in this cohort (25). Interestingly, this study also found a 22% higher rate of non-use of LLINs among males compared to females. This finding may have important implications for the multiple studies that have found gender differences in malaria susceptibility (26, 27). The objective monitoring used in this study represents a gender-neutral method, as compared to self-reports, of assessing LLIN use and may provide supportive evidence that socio-behavioral factors may put males at higher risk of malaria (28), although future studies would have to confirm these findings and rule out whether monitoring might differentially change LLIN use behaviors based on gender.

This study also provides evidence of the feasibility of objective monitoring of LLIN use. Previous studies have used these devices over shorter time periods (12, 15), and a goal of this study was to assess the feasibility of gathering data over longer times periods in field settings. In this study, using household visits every 2 weeks, ninety-six individuals of various ages from 20 households were successfully monitored over multiple months to obtain a large sample of LLIN use behaviors. Future work should leave these monitors in place through seasonal variations in malaria. In addition, the study provides evidence that remote bednet monitoring is most effective when combined with reported sleeping times, as the estimates of *Anopheles* exposure were similar when using self-reported bedtimes compared to using SmartNet data alone (Figure 7). The combination of both sleeping times and SmartNet monitoring provided the most plausible results and the richest understanding of *Anopheles* exposure in relation to LLIN use. Finally, in this study, the low incidence of malaria after years of IRS precluded the assessment of how LLIN use affects clinical malaria outcomes. Future work in higher transmission settings could tie LLIN use more directly to metrics of malaria infection and disease.

The version of the SmartNet technology in this study uses conductive fabric to identify whether a bednet is up or down and was determined by visual observation in this field setting to be 88% accurate. As was found in pilot studies, SmartNet tends to be more accurate at classifying LLINs that are unfurled than folded up (11). Newer developments in monitoring technologies, such as the use of accelerometers and machine learning algorithms, suggest that objective monitors can provide up to 96% accuracy and may also provide additional information about entries/exits from unfurled LLINs that may be relevant to malaria risk (29).

Compared to cohort individuals who were not monitored by SmartNet, either in the same households or in other households, there was much higher reported LLIN use in monitored individuals after SmartNet deployment, suggesting that objective monitoring itself may increase LLIN use. Nevertheless, the rate of non-use increased steadily over time in the monitored group, from 3.3% in the first month to 19% in the fourth and fifth month. This could represent a waning of this monitoring effect and a reversion to more typical use patterns, or it could reflect a response to seasonal fluctuations in mosquito density. Monitoring over longer time periods, through multiple seasonal peaks in mosquito exposure, would help define the degree to which objective monitoring itself impacts LLIN use.

There were multiple potential limitations in this study. Objective bednet use monitoring was not 100% accurate in this study. While SmartNet is arguably more accurate than self-reporting methods, there is still the potential for inaccuracy and bias with a less than perfect gold standard. Nevertheless, SmartNet inherently tends to over-estimate LLIN use (unfurled LLINs), so measures of LLIN non-use in this study are likely an underestimate from actual practice. In order to obtain data about actively used LLINs, the households chosen for SmartNet enrollment, and the sleeping areas receiving SmartNet monitors, were those already more likely to use LLINs. As a result, conclusions are not representative of the entire cohort nor of the population in the study site as a whole. The estimates of hourly mosquito exposure in this study were derived from HLC measures of indoor biting mosquitoes only and were performed the year prior to the study. As there was no available data on whether individuals were indoors or outdoors before their bedtimes, it was decided to use indoor measures of hourly exposure for the entire cohort. The sensitivity analysis exploring an extreme estimate of outdoor exposure showed even less protection from LLINs, so the adopted method is likely a conservative estimate. The HLCs were also not contemporaneous with the SmartNet study activities. However, the HLC activities were stopped in 2018 after they were found to produce little variation from previous years and this study attempted to account for potential seasonal differences by using the HLC data from the months corresponding to the SmartNet study in calculating the distribution of mosquitoes. The timing of captures was slightly different, as HLCs were performed from 6 p.m. to 6 a.m., but the CDC LTs were placed from 7 p.m. to 7 a.m. The observation period for SmartNet was from 6 p.m. to 6 a.m. to match with the hourly probabilities of exposure from the HLCs. Since CDC LTs are a general measure of the density of female *Anopheles* mosquitoes and this was applied across the whole population, this slight difference is unlikely to significantly affect the study results. Finally, mosquito density and reported bedtimes were measured every 2 weeks but SmartNet provides nightly data. Thus, nightly estimates of mosquito exposure and bedtimes were imputed from the most recent measured value for each individual. These methods could produce inaccuracies, but would not be expected to be systematically biased when applied equally across the entire study population.

## Conclusion

Objective monitors are accurate and can feasibly be deployed to obtain data about LLIN use. Despite high rates of reported LLIN use, LLINs provided protection from only an estimated 50% of female *Anopheles* mosquito exposure in this cohort and this protective capacity appeared to decrease with increasing age, although the study lacked adequate power to conclude that there was a statistically significant difference between age groups. These findings point out the importance of considering the dynamics between mosquito exposure and human behaviors in assessing malaria risk and prevention strategies. Taken together, the various components of this study demonstrate the power of objective monitoring to produce a deeper understanding of how LLINs are used and quantify their role in the prevention of malaria.

## Data availability statement

The raw data supporting the conclusions of this article will be made available by the authors, without undue reservation.

## Ethics statement

The studies involving human participants were reviewed and approved by Uganda National Council for Science and Technology (UNCST), Makerere University School of Medicine Research Ethics Committee, Mulago Hospital Research and Ethics Committee, University of California, San Francisco Committee for Human Research, London School of Hygiene and Tropical Medicine Ethics Committee. Written informed consent to participate in this study was provided by the participants' legal guardian/next of kin.

## Author contributions

PK and GD: conceptualization, funding acquisition, visualization, and writing—original draft. JR, AM, GO, PK, and JAR: data curation and investigation. PK, GD, KW, and AR: formal analysis. PK, GD, CD, KW, and AR: methodology. JR, AM, and GO: project administration. JR, GO, PK, EA, JN, and MK: supervision. JR and PK: validation. All authors provided critical feedback and helped shape the research, analysis, and manuscript. All authors contributed to the article and approved the submitted version.

## Funding

This work was supported by the National Institutes of Health as part of the International Centers of Excellence in Malaria Research (ICEMR) program (U19AI089674) and Career Mentored Award (PJK; K23AI139364). EA was supported by the Fogarty International Center of the National Institutes of Health (D43TW010526). JN was supported by the Fogarty International Center (Emerging Global Leader Award; K43TW010365). Opportunity Solutions International supplied the funds for the SmartNet devices used in the study. The funders had no role in the study design, data collection and analysis, decision to publish or preparation of the manuscript.

## Conflict of interest

SmartNet was invented by PJK who co-owns intellectual property in SmartNet. PJK is also a Co-founder and Director (unpaid) of the 501(c)3 non-profit organization Opportunity Solutions International (www.opportunitysolutions.org) which funded part of this work. Authors JR, GO, PK, JAR, EA, JN, and MK were employed by Infectious Diseases Research Collaboration.

## Publisher's note

All claims expressed in this article are solely those of the authors and do not necessarily represent those of their affiliated organizations, or those of the publisher, the editors and the reviewers. Any product that may be evaluated in this article, or claim that may be made by its manufacturer, is not guaranteed or endorsed by the publisher.

## Author disclaimer

The content is solely the responsibility of the authors and does not necessarily represent the official views of the National Institutes of Health.

## Abbreviations

CDC LT: Centers for Disease Control light trap
CI: confidence interval
HBR: Human biting rate
HLC: human landing catches
LLIN: long-lasting insecticide treated bednet
IRS: indoor residual spraying.
