# Supplementary material for: Remote bednet use monitoring to describe patterns of use and exposure to female Anopheles mosquitoes in an Ugandan cohort
Source: Front Epidemiol. 2022 Aug 4;2:934557. doi: 10.3389/fepid.2022.934557 (PMC10583855; doi:10.3389/fepid.2022.934557)
Supplement: Supplementary file 1 [file Table_1.DOCX]

**Additional file 1**

**Figure S1: Distribution of mosquitoes from human landing catches based on 8pm bedtime.** Data from 48 indoor and outdoor catches performed 6pm to 6am from May through October 2018 in 8 randomly selected households geographically proximate to study households. Hourly exposure is assumed to be equivalent to outdoor catches prior to bedtime and indoor catches after bedtime.

**Figure S2: Distribution of mosquitoes from human landing catches based on 9pm bedtime.** Data from 48 indoor and outdoor catches performed 6pm to 6am from May through October 2018 in 8 randomly selected households geographically proximate to study households. Hourly exposure is assumed to be equivalent to outdoor catches prior to bedtime and indoor catches after bedtime. 

**Figure S3: Distribution of mosquitoes from human landing catches based on 10pm bedtime.** Data from 48 indoor and outdoor catches performed 6pm to 6am from May through October 2018 in 8 randomly selected households geographically proximate to study households. Hourly exposure is assumed to be equivalent to outdoor catches prior to bedtime and indoor catches after bedtime. 

**Figure S4: Estimated proportion of female** ***Anopheles*** **mosquito** **exposure averted from bednet use by measurement method.** Sample restricted to 392 nights with reported use and assessed over 95 participants with reported use data. Hourly probability and density adjusted for assumed outdoor exposure up until reported bedtime and indoor exposure after reported bedtime. Bars represent 95% confidence intervals around labeled means.
